# Expiratory ventilation assistance versus pressure-controlled ventilation with ambient oxygen in a hemorrhagic trauma model: a prehospital rescue option?

**DOI:** 10.1186/s40635-025-00742-y

**Published:** 2025-03-07

**Authors:** Tomas Karlsson, Jenny Gustavsson, Katrin Wellfelt, Mattias Günther

**Affiliations:** 1 Department of Clinical Science and Education, Section of Anesthesiology and Intensive Care, Karolinska Institutet, Sjukhusbacken 10, 11883 Stockholm, Sweden; 2 Rapid Response Car AISAB, Stockholm, Sweden; 3 Department of Neuroscience, Karolinska Institutet, Stockholm, Sweden

**Keywords:** Airway obstruction, Expiratory ventilation assistance, Porcine class III hemorrhage, Ventrain, Whole blood resuscitation

## Abstract

**Background:**

Prehospital airway management is critical for maintaining oxygenation after severe trauma hemorrhage. In cases of semi-obstructed airways, intubation with an endotracheal tube may fail, whereas a 14 French intubating catheter may provide an alternative for ventilation. Expiratory ventilation assistance (EVA) through such a catheter could serve as a prehospital rescue option, particularly when oxygen supply is limited. This study evaluates whether EVA with ambient air is sufficient to maintain oxygenation and compares its effectiveness with pressure-controlled ventilation (PCV).

**Methods:**

Twenty-three anesthetized swines (mean weight 58.3 kg, SD 4.6) were subjected to 32% blood volume hemorrhage and allocated to either EVA (*n* = 11) or PCV (*n* = 12). Historical data were used in the control group. Three phases were studied: 15 min without intervention (emulating initial prehospital care), 30 min of whole blood resuscitation, and 15 min post-resuscitation. Parameters including oxygen delivery (DO_2_), oxygen consumption (VO_2_), arterial saturation (SaO_2_), intratracheal pressures, and lactate levels were measured.

**Results:**

EVA and PCV demonstrated similar effectiveness in maintaining indexed DO_2_ (*p* = 0.114), VO_2_ (*p* = 0.325), oxygen extraction rate (*p* = 0.841), and SaO_2_ (*p* = 0.097). Intratracheal pressures were significantly lower with EVA (*p* < 0.0001). EVA maintained clinically sufficient oxygenation (PaO_2_ > 8.6 kPa) but PaCO_2_ levels increased compared with control. Lactate levels were significantly lower in the EVA group during resuscitation (3.1 mmol/L vs. 4.8 mmol/L, *p* = 0.032).

**Conclusion:**

Both EVA and PCV effectively maintained oxygen delivery and sufficient oxygenation after trauma hemorrhage and whole blood resuscitation. Lower intratracheal pressures and reduced lactate accumulation with EVA suggest it may be a viable prehospital rescue method, especially in scenarios with limited oxygen supply. Further investigation is warranted to optimize its application.

**Supplementary Information:**

The online version contains supplementary material available at 10.1186/s40635-025-00742-y.

## Introduction

Effective prehospital airway management is essential to maintain oxygenation after severe trauma hemorrhage [1], but the risk of management failure increases in the prehospital environment [2, 3]. Tracheal intubation is the gold standard, commonly facilitated by an adjunct such as an introducing catheter (a “bougie” with a central lumen for oxygenation), in emergent cases. The catheter is inserted into the trachea, which then guides the endotracheal tube (ETT) into correct position [4–6]. In pending airway obstruction, inserting an ETT over the catheter may be impossible, leaving the catheter as the only access to the airway. In total obstruction, the patient cannot be intubated nor oxygenated, a “cannot intubate cannot oxygenate” (CICO) situation, which occurs in 0.2–0.5% of trauma and emergency patients [7–9]. This is particularly challenging in the prehospital environment and may lead to death or anoxic brain damage [10]. Thus, the consequences are severe in trauma and critically ill patients with limited physiological reserves [6]. Under these circumstances, the last resort is to perform a surgical emergency front-of-neck access (eFONA), where the catheter is inserted into the trachea through a surgical opening to aid the insertion of the ETT [11, 12].

Ventilation through a catheter positioned in the trachea may be lifesaving but requires enough pressure of breathing gas to overcome the resistance of the narrow lumen. Additionally, insufficient exhalation may cause air trapping and barotrauma. Expiratory ventilation assistance (EVA), a ventilatory modality which utilizes the Venturi effect to deflate the lung through a narrow lumen, normally propelled by oxygen, may be achieved by a handheld device [13, 14]. However, it is not known whether EVA through an intubating catheter would be sufficient to maintain oxygenation and ventilation after trauma hemorrhage, and whether sufficient oxygenation would be maintained with 21% O_2_, which is particularly important in austere prehospital and military tactical environments with limited O_2_ supply [15]. EVA has previously been shown to improve oxygenation compared with conventional positive pressure ventilation [16]. Therefore, we investigated the feasibility of EVA with 21% O_2_, through a 14 French intubating catheter in a semi-occluded airway, after porcine class III hemorrhage (> 30% of total blood volume) and whole blood resuscitation. We compared EVA through an intubating catheter with pressure-controlled ventilation (PCV) through an ETT, in three clinically relevant prehospital phases and hypothesized that EVA would be more efficient in oxygen delivery and thus feasible as a prehospital rescue method.

## Materials and methods

The study was approved by, and conducted in accordance with, the Swedish regional ethics approval board for animal research (approval no 12578-2020), and the ARRIVE guidelines (supplementary file 1). The datasets are available from the corresponding author on reasonable request. The experiments were performed under veterinary supervision between 21 April 2021 and 24 February 2023. We compared EVA with a control group of twelve animals, of which ten had been subjected to an identical hemorrhage protocol in an identical setting in our laboratory, as previously described [17]. The two additional control group animals were incorporated into the trial to comply with the power calculation. This allowed for optimized and ethical utilization of animals in compliance with Reduce, described in the 3R of ethical animal research [18], by decreasing the total amount of animals needed to test the hypothesis. Twenty-three swines were allocated to either EVA (*n* = 11) or PCV (*n* = 12).

### Preparation

Crossbred male-specific pathogen free swines (Company Johansson, Stockholm Region, Sweden) with a mean weight 58.3 (4.6) kg were premedicated with 150 mg tiletamine/zolazepam (Zoletil 100 Vet) and 6 mg medetomidine (Cepetor Vet). After preoxygenation and assisted ventilation, anesthesia was induced in the right or left auricular vein, in a supine position on a standard operating table, with alfentanil 40 µg/kg and pentobarbital sodium 6 mg/kg. Tracheal intubation was performed with a custom-made Miller-type laryngoscope and a Frova intubating catheter (Cook Medicals, Bloomington, IN, USA), using a standard size 8 mm inner diameter (ID) ETT (Rüsch, Teleflex, Morrisville, NC, USA), cuffed to 50 cmH_2_O. The ETT was connected to the ventilator tubing with a double swivel connector, second orifice used for bronchoscopic measurements during set-up, positioning of ETT proximally to the upper lobe bronchus and placement of tubing for continuous intratracheal pressure, connected to a TSD160D pressure transducer (Biopac systems, Goleta, CA, USA). Anesthesia was maintained with ketamine 20 mg/kg/h, midazolam 0.04 mg/kg/h. A 500 mL bolus of Ringer´s Acetate was given for 30 min after the induction of anesthesia. The animals were ventilated with a Hamilton C2 (Hamilton Medical, Geneva, Switzerland) using pressure synchronized intermittent mandatory ventilation with initial settings PEEP 4, PIP 15 cm H_2_O, respiratory rate 12/min, trigger flow 5 L/min and FiO_2_ 21%. Settings were continuously adjusted according to a predefined ventilator weaning protocol (supplementary file 2), with ZEEP, PIP 13–15 cmH_2_O (to achieve tidal volumes of 6–8 ml/kg), mandatory respiratory rate 10–12 and trigger flow 1 L/min at baseline. PIP and respiratory rate were lowered in small increments to allow for spontaneous ventilation, alongside increased trigger flow, during hemorrhage phase. A 7.5 F pulmonary artery catheter (Edwards Lifescience, Irvine, CA, USA) was introduced in the surgically exposed right internal jugular vein and used for core temperature, central venous pressure, pulmonary artery pressure, cardiac output (CO) and mixed venous oxygen saturation (SvO_2_). The right brachial and femoral arteries were cannulated, ultra-sound assisted with a 20 G (Braun Medicals, Melsungen, Germany) and a 7 F catheter, respectively (Merit Medical, South Jordan, Utah, USA) and allowed for continuous blood pressure measurements, blood samples and blood removal. A twelve-lead ECG was connected, and a supra-pubic catheter was inserted.

### Intervention

**Fig. 1:**
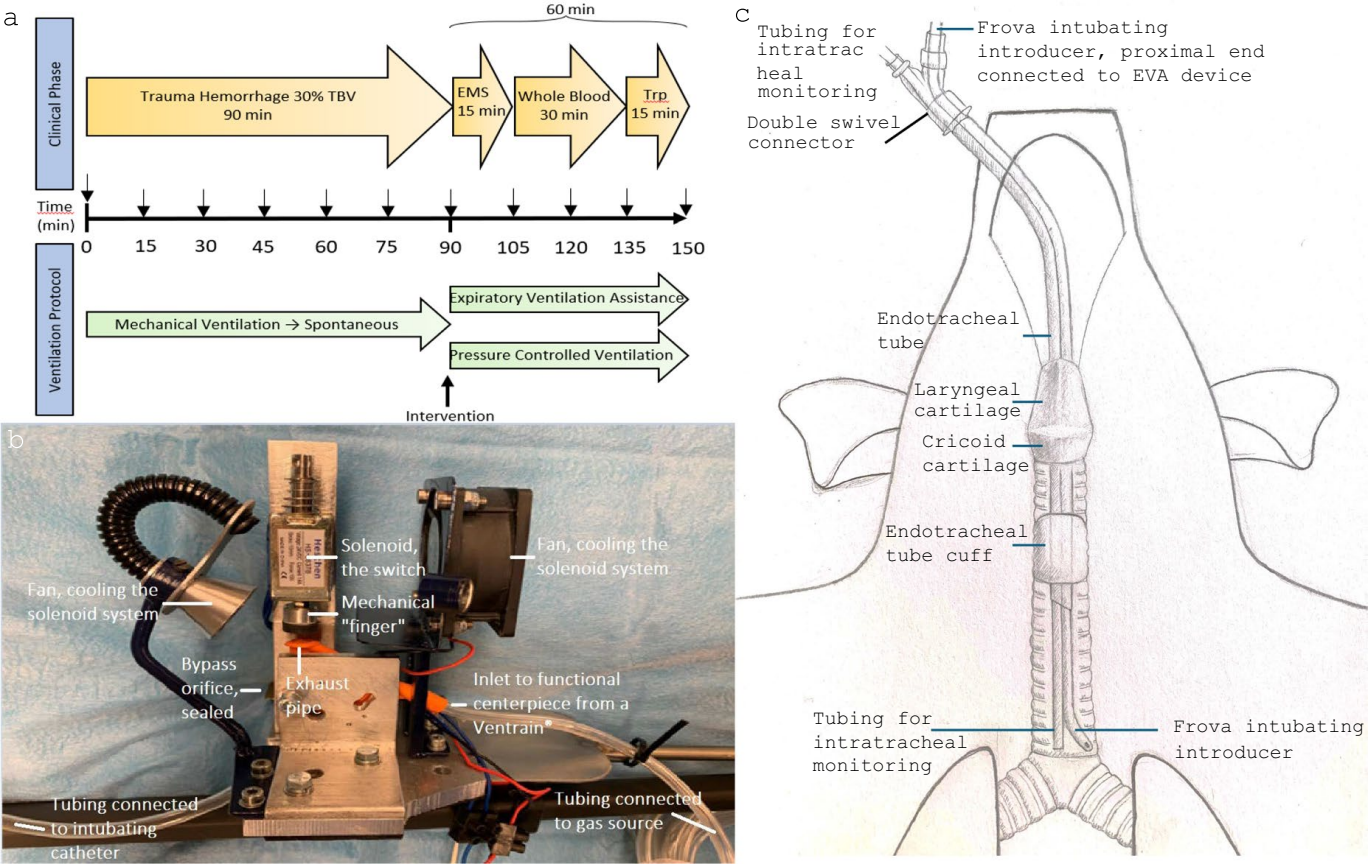
**a** Experimental set-up, describing the prehospital clinical phases and the ventilation protocol. Arrows are times of sampling. **b** Custom-made expiratory ventilation assistance (EVA) device with a mechanical “finger” and the functional centerpiece from a Ventrain®. **c** Drawing of experimental set-up, showing the location of tubing for intratracheal pressure monitoring and intubating introducer

**Fig. 2:**
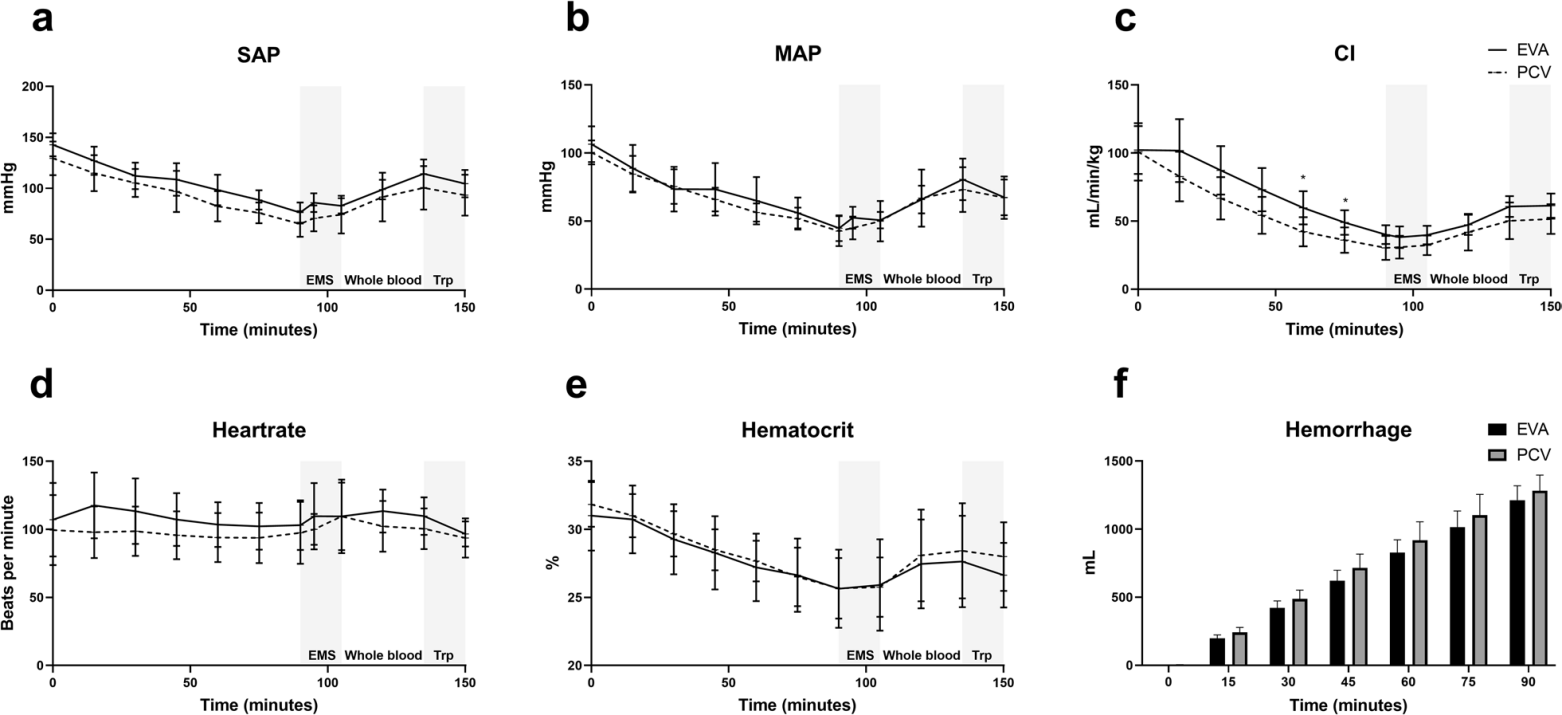
Circulatory effects. No differences were detected in **a** systolic arterial pressure (SAP) **b** mean arterial pressure (MAP) **c** cardiac index (CI) **d** heartrate **e** hematocrit or **f** hemorrhage between EVA and pressure-controlled ventilation (PCV). Mixed-effect model analysis and descriptive statistics (**f**). *EMS*: emergency medical service phase with initial intervention. Whole blood: transfusion phase. *Trp* transportation phase

Arterial blood gasses (pH, PaCO_2_, PaO_2_, lactate, arterial blood saturation (SaO_2_) and base excess, BE) were collected at baseline and then every 15 min for 150 min (GEM Premier 4000, Instrumentation Laboratories, Lexington, MA, USA). A controlled bleeding was accomplished by a peristaltic pump (Masterflex L/S, Cole Parmer, IL, USA). A mean 1249 (113) mL (SD), corresponding to 32% blood loss from an estimated total blood volume (TBV) of 67 mL/kg, was drawn for 90 min. The shed blood was stored in citrated bags (Fresenius Kabi, Uppsala, Sweden) and saved for subsequent autologous whole blood transfusion. A total of (standard deviation (SD)) 510 (55) mL (13% of TBV) were infused (Fig. 1a). No calcium was administered as calcium levels remained within limits. Inclusion criteria were SaO_2_ ≥ 80%, 100% spontaneously breathing with 3 cmH_2_O pressure support and 5 L/min trigger at intervention, uninhibited insertion of the intubating introducer, normoglycemia and sinus rhythm throughout the trial. A total of 25 were assessed for eligibility and 23 were included. One animal had a pneumothorax by accidental perforation of the trachea by the intubating introducer after resistance to insertion, and one had severe hypoglycemia, which would have confounded the results and were excluded. Both groups had an ETT left in situ to facilitate the introduction of the intubating introducer and to get the same semi-occlusion in all animals. In EVA, the ventilator tubing was disconnected from the ETT and the intubating introducer was inserted to a premeasured depth, one centimeter above the carina (Fig. 2a), and luer-lock connected to a custom-made construction, consisting of a solenoid acting as a switch with a central mechanical “finger”, intermittently sealing the exhaust-pipe hole of the functional centerpiece from a Ventrain (Ventinova Medical, Eindhoven, the Netherlands). The bypass orifice of the centerpiece was sealed (Fig. 1b). A fan was integrated in the construction to cool the solenoid. A gas flow of 15 L/min and an inspiration/expiration (I:E) ratio of 1:1 was set, with two seconds of inspiration and two of expiration, generating a tidal volume of 500 mL. The mechanical device was electronically steered with exact time intervals. Semi-occlusion was achieved in the intervention group through an intubating catheter (4.67 mm outer diameter (OD)) and tubing (4 mm OD) for intratracheal pressure measuring (supplementary file 3) located inside the cuffed (50 cmH_2_O seal-pressure) 8 mm ID ETT (Fig. 1c). Rocuronium bromide (total approximately 3 mg/kg) was administered to prevent spontaneous ventilation in both groups. After fifteen minutes of emergency medical service (EMS) intervention phase, whole blood transfusion (resuscitation phase) was started through an auricular vein, using standard clinical blood infusion sets, with a rate of approximately 17 mL/min for 30 min. The transport phase was the last 15 min after completion of transfusion, corresponding to the time required for transport from a remote location (Fig. 1a). After completion of the experiment, euthanization was achieved with 60 mL of pentobarbital sodium (Allfatal Vet 100 mg/mL).

### Calculations

Oxygen delivery (DO_2_) was calculated using: DO_2_ = CO x (1.34 × Hb x SaO_2_) + (PaO_2_ × 0.225), CO (L/min), Hb (g/L), SaO_2_ (% × 100^–1^), PaO_2_ (kPa), Hüfner´s constant = 1.34 (the volume (mL) of oxygen carried by 1 g of 100% saturated Hb) and the constant 0.225 (the amount (mL) of oxygen dissolved in 1 L of blood per kPa). Oxygen consumption (VO_2_) was calculated using: VO_2_ = CO (CaO_2_ -CvO_2_), with CaO_2_ = 1.34 × Hb x SaO_2_ and CvO_2_ = 1.34 × Hb x SvO_2_ [19, 20]. DO_2_I, VO_2_I and CI were indexed to the weight in kg.

### Statistical analyses

Statistical analyses were performed using GraphPad Prism version 10.2.1 (GraphPad Software, La Jolla, CA, USA). The primary outcome was DO_2_, and secondary outcomes were oxygenation, ventilation, intratracheal pressures and hemodynamic parameters. We performed an a priori power calculation based on DO_2_ 397 with mean (SD) 88 mL/min after whole blood resuscitation, using data from previous experiments [17]. An increase (effect size) of 25%, alpha 0.05 and 0.8 power gave *n* = 12. The power calculation was performed using https://clincalc.com/stats/samplesize.aspx. Data were tested for normality with Shapiro–Wilk test. All temporal data sets were analyzed with mixed effects model (REML), fixed effects (type III) instead of ANOVA, due to solitary missing values. Šídák´s multiple comparisons test was implemented where appropriate. Unpaired t-test was used to analyze delta change. Data were presented as mean (SD) and 95% confidence interval (95% CI). Error bars show the standard deviation. A *p* < 0.05 was considered significant. *p < 0.05, **p < 0.01, ***p < 0.005, ****p < 0.0001.

## Results

Before intervention (90 min), EVA and PCV were equal in SAP, MAP, CI, heartrate, hematocrit, and hemorrhage. There was a mean difference (MD) of 13.3 (95% CI − 5.7–32.2, *P* = 0.347) in SAP from the start. At 90 min, SAP was (SD) 76.1 (10.0) mmHg in EVA and 65.0 (12.6) in PCV (MD 11.1, 95% CI − 4.0–26.2, *P* = 0.292). CI was (SD) 40.1 (6.9) mL/min/kg and 30.4 (8.8), respectively (MD 9.7, 95% CI − 0.8–20.3, *P* = 0.086) but 101.8 (23.1) and 82.8 (18.2), respectively (MD 19.0, 95% CI − 9.3–47.3, *P* = 0.403) after 15 min (Fig. 2).

At start of the Emergency medical services (EMS) phase (90 min), DO_2_I were (SD) 5.0 (0.8) mL/min/kg in EVA vs 3.9 (1.1) in PCV (MD 1.1, 95% CI − 0.2–2.4, *P* = 0.144). At start of whole blood transfusion at 105 min, results showed 5.1 (0.9) vs 4.3 (0.9) (MD 0.9, 95% CI − 0.3–2.1, *P* = 0.314). At start of transport phase (135 min), results showed 7.5 (1.3) vs 6.7 (1.5) (MD 0.8, 95% CI − 1.1–2.6, *P* = 0.921). No differences were detected between groups in any phase with mixed-effect analysis (*P* = 0.114).

No differences were detected in VO_2_I (*P* = 0.325). VO_2_I were at start of the hemorrhage (SD) 4.4 (0.6) mL/min/kg vs 4.6 (0.7) (MD − 0.2, 95% CI − 1.1–0.7, *P* > 0.999). At start of the EMS phase (90 min), values were 2.9 (0.8) vs 2.3 (0.9) (MD 0.7, 95% CI − 0.5–1.8, *P* = 0.580), which was the lowest point.

No difference was seen in O_2_ER (*P* = 0.841). At the start, EVA vs PCV were 30.6% (3.6%) vs 33.1% (8.6%) (MD − 2.5%, 95% CI − 11.5–6.5%, *P* = 0.994). The highest O_2_ER occurred at start of whole blood resuscitation and were 62.1% (17.0%) vs 60.7% (15.6%) respectively (MD 1.3%, 95% CI − 20.4–23.0%, *P* > 0.999).

At completion, PaO_2_ was (SD) 9.2 (1.2) kPa in EVA and 11.9 (2.5) kPa in PCV (MD − 2.7, 95% CI − 5.3 to − 0.04, *P* = 0.045). At all other timepoints of the three phases, PaO_2_ was above (SD) 8.6 (1.0) kPa in EVA with no difference between the groups. Arterial saturation remained above (SD) 85.8 (7.6) % in EVA and 90.9 (9.3) % in PCV throughout the trial without any difference (*P* = 0.097).

**Fig. 3:**
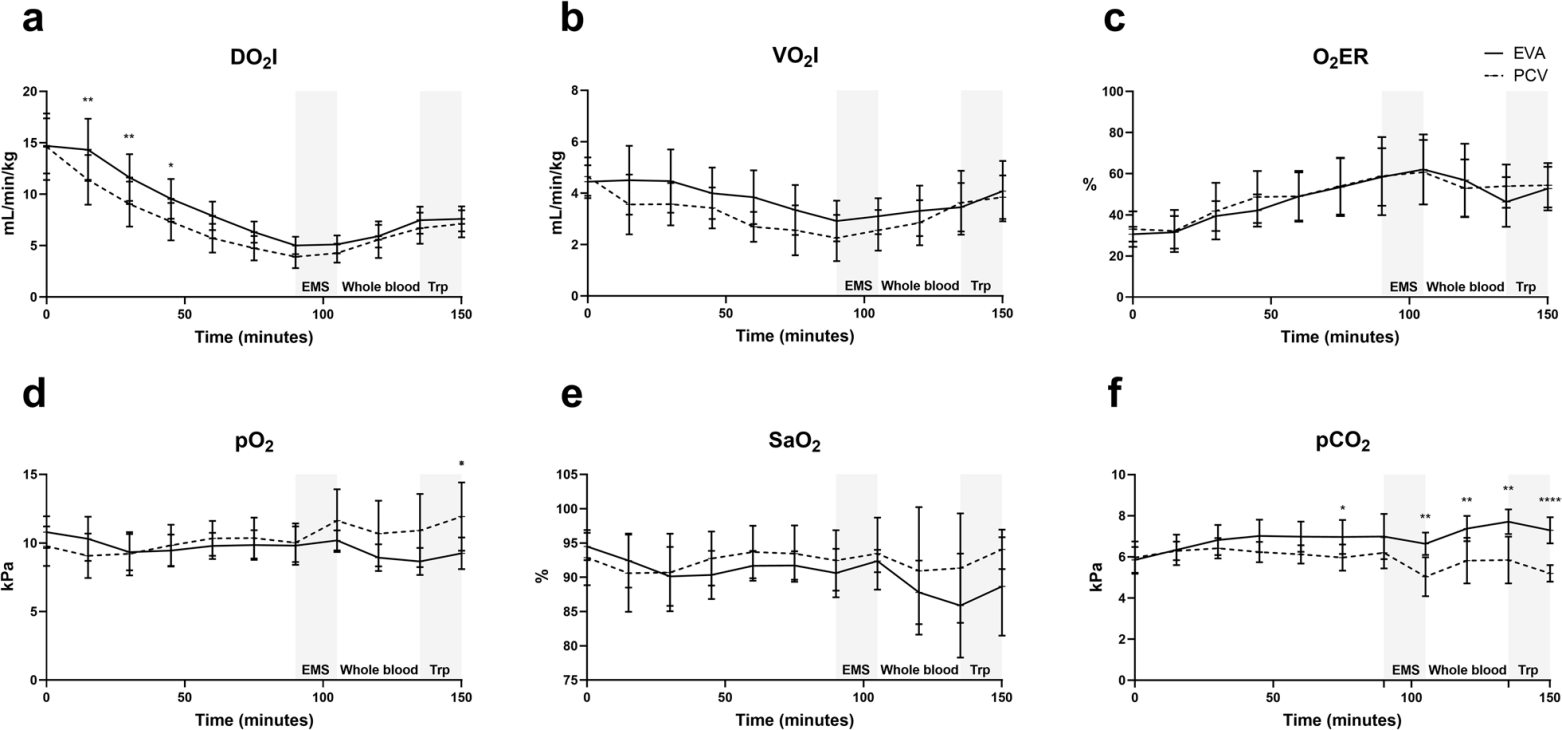
Oxygenation and ventilation effects. No differences were detected in **a** indexed oxygen delivery (DO_2_I) **b** indexed oxygen consumption (VO_2_I) **c** oxygen extraction rate (O_2_ER) **d** partial pressure of oxygen (PaO_2_) (except at 150 min) or **e** arterial saturation (SaO_2_) between EVA and PCV after intervention but **f** difference was detected in partial pressure of carbon dioxide (PaCO_2_) between EVA and PCV, with a maximum change of 0.7 kPa (10%) in EVA. Mixed-effect model analysis. *EMS* emergency medical service phase with initial intervention. *Whole blood* transfusion phase. *Trp* transportation phase

PaCO_2_ was (SD) 7.0 (1.1) kPa in EVA and 6.2 (0.8) kPa in PCV (MD 0.8, 95% CI − 0.5–2.1, *P* = 0.509) at end of hemorrhage. At the start of the transfusion, PaCO_2_ was (SD) 6.6 (0.5) kPa and 5.0 (1.0) kPa (MD 1.6, 95% CI 0.6–2.6, *P* = 0.001). In EVA, PaCO_2_ peaked at (SD) 7.7 (0.6) kPa after the end of transfusion and at completion, (SD) 7.3 (0.6) kPa and 5.2 (0.4) kPa (MD 2.1, 95% CI 1.4–2.8, *P* < 0.0001). No difference in PaCO_2_ was seen in EVA, at completion compared with end of hemorrhage (*P* = 0.439) (Fig. 3).

**Fig. 4:**
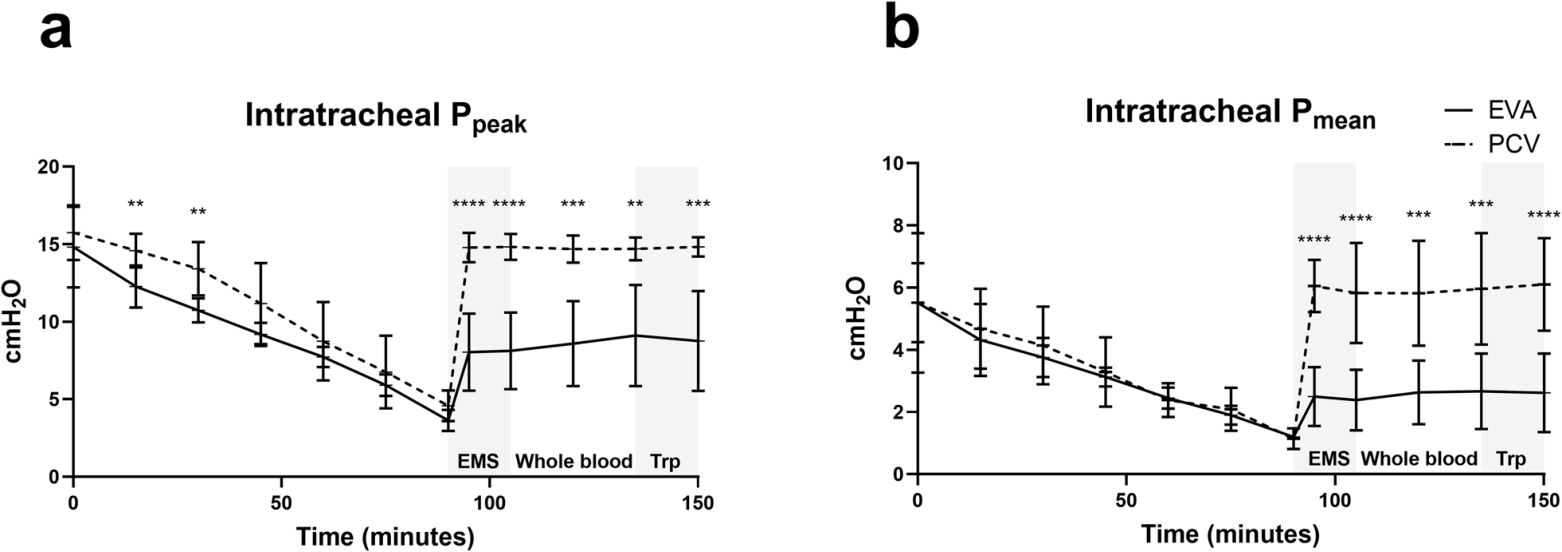
Airway pressure effects. After intervention intratracheal **a** peak pressures and **b** mean pressures were significantly lower in EVA than PCV. Mixed-effect model analysis. *EMS*: emergency medical service phase with initial intervention. *Whole blood* transfusion phase. *Trp* transportation phase

Intratracheal peak pressures varied between (SD) 8.1 (2.5) cmH_2_O and 9.1 (3.3) in EVA and 14.7 (0.9) cmH_2_O and 14.8 (0.8) (*P* = 0.002 to *P* < 0.0001) in PCV and mean pressures were between 2.4 (1.0) cmH_2_O and 2.7 (1.2) in EVA and 5.8 (1.7) cmH_2_O and 6.1 (1.5) (*P* = 0.0006 to *P* < 0.0001) in PCV from EMS phase until completion. Both pressures were significantly lower in EVA vs PCV at all timepoints (Fig. 4).

**Fig. 5:**
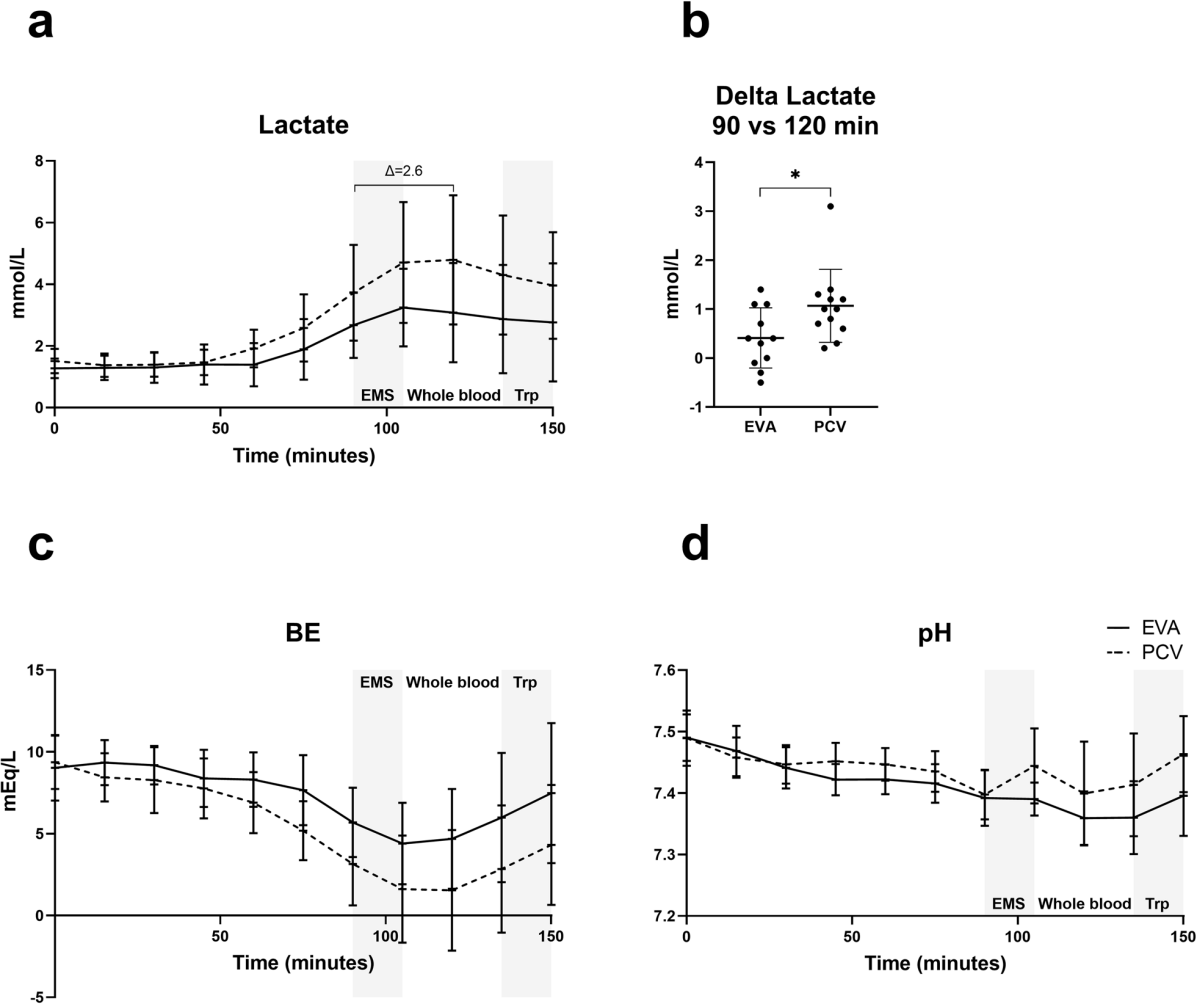
Metabolic effects. **a** A relative increase (delta) in lactate of 2.6 times in PCV vs EVA **b** at start of intervention compared with 15 min into whole blood transfusion and equal **c** base excess (BE) and **d** pH. Mixed-effect model analysis and unpaired *t*-test (**b**). *EMS*: emergency medical service phase with initial intervention. *Whole blood* transfusion phase. *Trp* transportation phase

Lactate levels increased, starting from 45–60 min of bleeding until the start of transfusion at 105 min in EVA and 15 min into transfusion in PCV. In EVA, the lactate peaked at (SD) 3.2 (1.3) mmol/L at 105 min and in PCV at 4.8 (2.1) mmol/L at 120 min. Lactate increased more in PCV compared with EVA at 120 min (*P* = 0.032) with a relative increase, delta, of 2.6 (mean 1.1 vs mean 0.4). No differences were detected in base excess or pH (Fig. 5).

## Discussion

In this study, we show that EVA through an intubating catheter in a semi-occluded airway was feasible with 21% O_2_ and maintained clinically sufficient DO_2_ during whole blood resuscitation. Oxygenation and ventilation were clinically sufficient albeit in the lowest acceptable range.

The most common errors in prehospital trauma care are related to airway management [21]. The prehospital environment is challenging and may require stabilization of severe injuries in austere environments. Therefore, improved methods of airway management before the patient arrives in the emergency department may decrease potentially preventable trauma deaths, i.e., deaths that could be avoided if optimal care had been delivered.

EVA is based on the concept of Bernoulli’s principle. Case reports suggest that the method may be effective as a rescue in clinical situations [22, 23], and experimental studies in pigs have shown that oxygenation may be maintained after airway obstruction, using 100% O_2_ [13, 14]. We investigated the feasibility of EVA with 21% O_2_, after severe trauma hemorrhage, for three reasons. First, the oxygen supply is in principle almost always limited outside of hospitals. Second, severe trauma hemorrhage increases the oxygen demand, which may limit the applicability of EVA ventilation. Third, 100% O_2_ would generate hyperoxemia which could conceal differences between groups and cause an incorrect hypothesis testing. We studied three phases: (1) During 15 min without intervention, illustrating the first minutes on scene by the EMS. (2) During 30 min of whole blood transfusion, defined as the time required for one unit of blood to be transfused through an intraosseous needle and a pressure bag (17 mL/min) [24]. (3) During 15 min after infusion, illustrating the transport time to a receiving hospital from remote areas [25, 26].

The intention was to achieve a severe but not fatal hemorrhage in combination with a semi-obstructed airway. For technical reasons, the swines were first intubated, and then weaned from the ventilator to create spontaneous breathing before the intervention. Therefore, the hemorrhage was a slow and constant trauma hemorrhage.

Critical DO_2_ (when tissue hypoxia occurs), defined as increased lactate and > 2 mmol/L, occurred at a DO_2_ of 5–6 mL/min/kg, approximately 65–80 min from the start of hemorrhage. At this point, VO_2_ becomes dependent on DO_2_. Thus, below this level, the cells produce an increasing amount of anaerobic energy to meet the metabolic demand, producing lactate [20, 27–29]. The critical DO_2_ of 5–6 mL/min/kg occurred similarly to our previous work in swine [17] and the work of others, in non-human primates and humans [20, 28, 30]. After completion of whole blood resuscitation, both groups restored DO_2_ levels above critical. Lactate decreased at the start of resuscitation in EVA, in comparison to 15 min of resuscitation in PCV, suggesting that EVA was more energy efficient. However, the EVA group started from a lower lactate level. Therefore, we calculated the lactate changes in each group at different timepoints, compared the two groups and confirmed a difference in increase. The lactate level in EVA indicated a higher systemic ratio of aerobic to anaerobic metabolism. This was possibly due to better end organ perfusion in EVA caused by the lower intratracheal pressures, thus better preload and CO, even though there were no detectable differences in hemodynamics. A previous publication with a similar ventilation method in an ARDS model, also described decreased lactate levels compared with PCV, possibly due to interaction of slow intrathoracic pressure changes with the cardiac cycle in flow-controlled ventilation compared with the rapid changes in PCV [31]. Lower energy dissipation of EVA in the lung and chest compared with PCV may have been an alternative explanation or a contributable factor [32]. This should be the focus of further investigations.

Arterial saturation did not decrease below 85% at any time in EVA, and there was no difference in comparison to PCV. No difference was seen in PaO_2_ except from a single time point at 150 min. PaO_2_ remained above 8.6 kPa in EVA throughout the observation time. We believe that the lowest saturation and PaO_2_ in EVA, seen at completion of transfusion, were still clinically sufficient, because DO_2_ was above a critical threshold and lactate decreased. In contrast, 100% O_2_ causes supranormal PaO_2_ [14].

Ventilation by EVA in semi-obstructed airways caused hypoventilation with a maximum increase in PaCO_2_ of only 0.7 kPa and remained within 10% from end of hemorrhage throughout the hour of intervention. In a previous pig study, EVA with a transtracheal catheter functioned better in a totally occluded airway compared with semi-occlusion and no occlusion. Semi-occlusion decreased PaCO_2_ during six minutes of ventilation [14]. Similarly, we found that the first 15 min of EVA without whole blood transfusion decreased PaCO_2_ albeit not significantly. A minute ventilation of 7.5 L/min with a gas flow of 15 L/min and an I:E 1:1 can be achieved with EVA through a 100 cm long airway exchange catheter with 3 mm inner diameter in vitro, when the Venturi effect works under optimal conditions in total airway obstruction [33]. Hence, the minute ventilation is a limitation for this method of ventilation unless flow is increased.

We detected no difference in circulation. Both groups maintained comparable cardiac output and blood pressures. After 15 min, and resuscitation with approximately 20% of lost blood volume or 250 mL, both groups reached a mean MAP of approximately 65 mmHg. This was comparable to our previous study with spontaneously breathing animals [17]. Thus, the effect of EVA on circulation was limited, if any. Similarly, partial obstruction does not affect blood pressure negatively in animals without hemorrhage [14].

The study has some limitations to be discussed. First, we compared EVA with a group of animals receiving PCV, in an identical setting from a previous trial, and the study was therefore not randomized. This increased the risk of selection bias. However, no differences between groups in any primary or secondary parameters were detected at the time of intervention, which lowered the risk for diverging parameters affecting the results. Second, the control group was ventilated with PCV in a closed circle system and EVA in a semi-obstructed system which may yield different ventilatory conditions because EVA had an inherent leakage. However, the performance of EVA was intended to be compared with gold standard ventilation (mechanical ventilation). Third, the hemorrhage was stopped before the resuscitation started, which may not always be the case in clinical trauma. Fourth, the model produced a controlled hemorrhage for 90 min and extrapolation to a short and uncontrolled hemorrhage should be done with caution. Fifth, the animals were anesthetized with ketamine and midazolam during the experiments. For this reason, we could not investigate the effects of prehospital anesthesia induction. Sixth, this was an animal model with a young and healthy population, and extrapolation to humans and specifically an elderly trauma population should be done with caution. Seventh, two animals in the EVA group were excluded due to events unrelated to the study´s primary endpoint, resulting in *n* = 11 in the EVA group. Although this increased the risk of a type II error, a post hoc analysis confirmed no differences between the groups and standard deviations remained comparable indicating that statistical power was only marginally affected. Therefore, we deemed the likelihood of a type II error to be low and the primary outcome would only be marginally affected with additional inclusions.

## Conclusion

Both EVA and PCV effectively maintained oxygen delivery and sufficient oxygenation after trauma hemorrhage and whole blood resuscitation. Lower intratracheal pressures and reduced lactate accumulation with EVA suggest it may be a viable prehospital rescue method, especially in scenarios with limited oxygen supply. Further investigation is warranted to optimize its application.

## Supplementary Information

Supplementary Material 1.

Supplementary Material 2.

Supplementary Material 3.

## Data Availability

The datasets are available from the corresponding author on reasonable request.

## Abbreviations

CaO_2_: Oxygen content of arterial blood
CI: Cardiac index
CICO: Cannot intubate, cannot oxygenate
CO: Cardiac output
CvO_2_: Oxygen content of venous blood
DO_2_: Oxygen delivery
DO_2_I: Indexed oxygen delivery
eFONA: Emergency front-of-neck access
EMS: Emergency medical services
ETT: Endotracheal tube
EVA: Expiratory ventilation assistance
ID: Inner diameter
I:E ratio: Inspiratory/expiratory ratio
OD: Outer diameter
O_2_ER: Oxygen extraction ratio
PaCO_2_: Arterial partial pressure of carbon dioxide
PaO_2_: Arterial partial pressure of oxygen
PCV: Pressure-controlled ventilation
VO_2_: Oxygen consumption
VO_2_I: Indexed oxygen consumption
