# Supplementary material for: Expiratory ventilation assistance versus pressure-controlled ventilation with ambient oxygen in a hemorrhagic trauma model: a prehospital rescue option?
Source: Intensive Care Med Exp. 2025 Mar 7;13:31. doi: 10.1186/s40635-025-00742-y (PMC11889277; doi:10.1186/s40635-025-00742-y)
Supplement: Supplementary file 2 — Supplementary Material 2. [file 40635_2025_742_MOESM2_ESM.docx]

**Weaning protocol**

In order to enable spontaneous breathing to return as quickly as possible after induction of the pig, this protocol shall be initiated as soon as possible. At randomization (T90, after 90 min), it is mandatory that the pig has regained consistent spontaneous breathing, otherwise the swine will be excluded.

1. After intubation, the following default settings are set: Pressure synchronized intermittent mandatory ventilation (PSIMV+), Peak inspiratory pressure (PIP) 15 cmH_2_O, Respiratory rate (RR) 12, Positive end-expiratory pressure (PEEP) 4 cmH_2_O, Trigger flow 5 L/min. PEEP 4 is set initially, to some extent counteract to the inevitable atelectasis development after induction. Respiratory rate is adjusted to achieve hypoventilation, etCO_2_ > 5.3 kPa. PEEP is gradually lowered during the set-up phase (cannulation etc)
2. The trigger flow is set by default to a value of 5 L/min at start-up, this is changed to 1 L/min. This is to ensure that there are self-triggered breaths as soon as possible after intubation and before randomization.
3. When the set-up phase is complete (after approximately 90 minutes), PEEP should be down to 0 cmH_2_O.
4. Gradually but slowly, lower the mandatory respiratory rate with 1-2 breaths from the initial rate to a respiratory rate of 5 (lowest value) to increase the PaCO_2_, and stimulate spontaneous triggering. When the pig has started to breathe with a rate that exceeds the mandatory rate, this step is not necessary. This step must be paused if the saturation becomes inadequate.
5. Gradually lower the PIP (pressure support). Aim at a tidal volume between 6-8 ml/kg at baseline. After hemorrhage start this should be done in small increments to reduce the formation of atelectasis. Reduce from 15 to 13 cmH_2_O, if not already done, and then reduced by 1 cmH_2_O approximately every 3-5 min.
6. The minimum pressure support is 3 cmH_2_O, which corresponds to the tube compensation in the breathing circuit.
7. Trigger flow is gradually increased after the return of spontaneous breathing and must be steady at 5 L/min before randomization.
