# Supplementary material for: Expiratory ventilation assistance versus pressure-controlled ventilation with ambient oxygen in a hemorrhagic trauma model: a prehospital rescue option?
Source: Intensive Care Med Exp. 2025 Mar 7;13:31. doi: 10.1186/s40635-025-00742-y (PMC11889277; doi:10.1186/s40635-025-00742-y)
Supplement: Supplementary file 3 — Supplementary Material 3. [file 40635_2025_742_MOESM3_ESM.docx]

**Supplementary file 3**

Example of intratracheal pressure trace showing pressures increasing in a supralinear and decreasing in a sublinear pattern with 8 cmH_2_O of peak pressure, -3 cmH_2_O of minimal pressure and in a) 12 seconds, b) and c) 60 seconds between vertical lines. In c) the end of the pressure trace from the pressure supported breathing in the ventilator, as well as the effect of the administration of rocuronium on the breathing, is seen, before initiation of expiratory ventilation assistance (EVA).

a


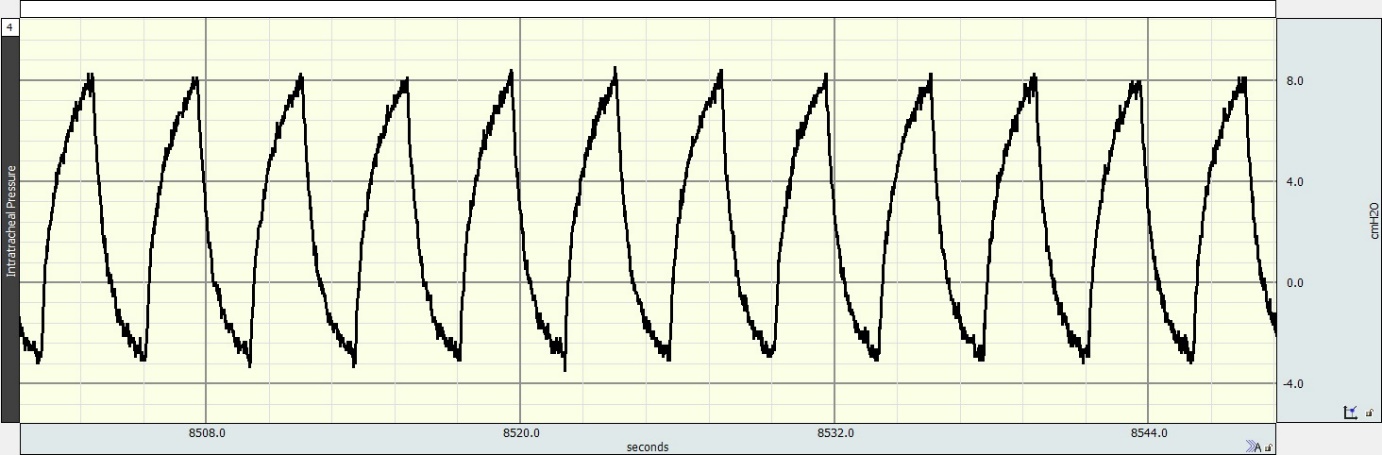

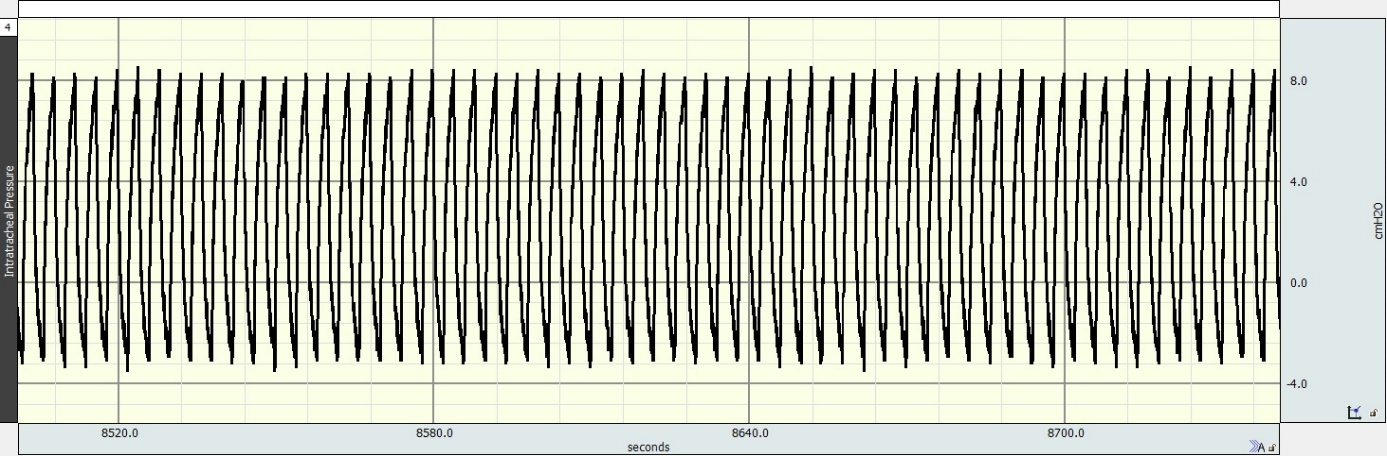

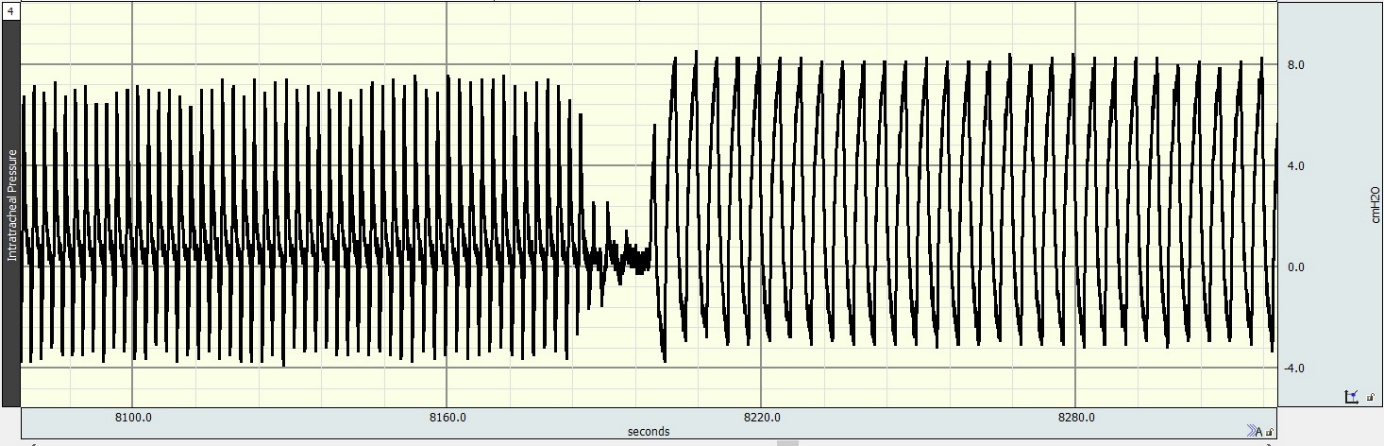


c

b
